# Eligible Pharmaceutical Forms for the Administration of Medicines

**Published:** 1862-03

**Authors:** Jno. Maynard Woodworth


ARTICLE IX.

### ELIGIBLE PHARMACEUTICAL FORMS FOR THE ADMINISTRATION OF MEDICINES.

AN INAUGURAL THESIS, BY JNO. MAYNARD WOODWORTH, M.D.

Since the very object of the profession is to alleviate human suffering, it may seem strange that the physician should unnecessarily augment it; but that he does so, almost as often as he writes a prescription, cannot be doubted. Most physicians look upon the disagreeable dose they often prescribe, as a matter of course, an evil not to be avoided; and it is to be regretted that most practitioners think if their medicines only cure the disease, it matters little in what way the dose is administered. I believe that disgust for disagreeable things can be cultivated; and this ingredient of human nature seems peculiarly susceptible to repulsive medicines. The child never forgets the taste of castor-oil or senna-tea; years after, the sight or smell—and to some the mere mention of the name, will excite nausea. This is no fancied sensation, but a real one, and I apprehend that upon this fact more than any other, depends the idiosyncrasy of some persons to certain medicines.

That the bad effects, produced by the disagreeable sight and disgusting taste of some medicines, often defeats the purpose for which they were given, is a fact that cannot be questioned; but the evil does not stop here, for so great is the dislike for the bitter dose, that some persons allow their sense of taste to govern them altogether, and they patronize that unmitigated humbug—Homœopathy.

All that Homœopathy can claim in its favor, is, first:—its medicines are agreeable; and, second:—it satisfies the mind that something is being done,—providing the patient has faith in it. These two, together with a kind of morbid inclination in men, and particularly in women, to run after every new thing, are the only reasons why Homœopathy is at all tolerated in the community.

This fungus growth of the healing art, will however, not exist in vain. The butterflies and insects that are born into the world full grown, live only a few hours, to propagate their species, and when their life-work is done, they die. So with Homœopathy; when we have applied the principle of agreeable exhibition of medicines, which is its only life-spark, it will die a natural death.

Great improvements have been made in the last hundred years—but the fathers have sought most after remedial agents, and have left for us to discover the most eligible forms for their administration. One hundred and fifty years ago, distilled vipers, scorpions, frogs, worms, little dogs, lizards, mice, their teeth, skulls, liver, tongues, tails, claws, etc.; feathers and brains of birds, shavings of men's skulls put to violent death, precious stones and metals, together with the leaves and roots of some of our medicinal plants, and a few chemicals, constituted a fair type of the pharmacopea. From these the physician made his prescription, sometimes incorporating over sixty ingredients in a single powder or mixture.

Notwithstanding the doctors of the last century prescribed so many disagreeable things, still they had an eye to please, as will be seen from the following prescription:—

℞. Oriental Pearls prepared.
Deer's Heart-bone aa ʒi.
Leaves of Gold and
Silver, - - aa ℈i.

and a dozen other ingredients, all were finely powdered, except the gold and silver, which were cut in little pieces for beauty's sake. This powder was said to create cheerfulness, to bring a good color into the face, and to strengthen and comfort the heart; an effect which I have no doubt it would have at the present day.

Improvement in the preparation of medicines is left too exclusively to the pharmaceutist.

The physician who has access to the sick chamber, and watches the effect of remedies at the bed-side of his patients, is surely the most capable of judging in what form a medicine should be prepared to render it the most agreeable. Mixtures with a repulsive taste or smell, or with a sediment in the bottom of the bottle, should never be allowed in the sick chamber. All that is disagreeable in taste or smell should be covered by the addition of some agreeable adjuvant, and when possible, they should be of some bright color; care even should be taken to have the label pasted squarely on the bottle. As small a matter as a crooked label may seem to be, there are persons, who when irritable and weak, would be worked into quite a fever by seeing such evidence of want of precision before them all day.

Officinal vinegars and dilute mineral acids, are rendered more agreeable by the addition of a small quantity of an alcoholic solution of some essential oil, as lemon, pimento, wintergreen, ceylon cinnamon, etc. Dilute phosphoric acid should have added to it a solution of the essential oil of sweet almonds. Compound tincture of cardamom and concentrated infusion of rose, are excellent additions to medicated acids or vinegars—covering a portion of their taste and imparting an agreeable color. Lactic acid, which is used by many physicians as a remedy for dyspepsia and indigestion, is made into an agreeable drink with water, sugar, and essence of lemon.

Jellies of copaiba or the fixed oils, as cod-liver and castor-oil, are agreeable; and may be prepared thus:—Take of the copaiba or fixed oil, four ounces; honey, two ounces; powdered gum arabic, one ounce; Russian isinglass, three drachms; orange-flower water, three ounces. Dissolve the isinglass by aid of heat in two ounces of the orange-flower water. Triturate the other ingredients with the remainder of the orange-flower water in a warmed mortar and form an emulsion by adding the solution of isinglass; stir until nearly cool, and set aside to gelatinize. It is best flavored with a little oil of bitter almonds. The fishy taste of cod-liver oil, which renders this favorite medicine absolutely impossible to be retained in the stomach of some persons, may be completely destroyed by the addition of a few drops of the essential oil of bitter almonds, and at the same time, the prussic acid of the oil of bitter almonds acts as a sedative to allay the cough of the patient. Chewing orange peel or burnt coffee before taking cod-liver oil will be found of advantage. A very agreeable emulsion of cod-liver oil may be prepared with carbonate of potassa, sugar, and cherry laurel or orange-flower water. Emulsions should be perfectly white, and agreeably flavored. Milk is a natural emulsion, in which butter is suspended in an aqueous liquid, by albumen and caseine. I believe that nature has chosen the best substances to form an emulsion; I therefore see no good reason why albumen should not, in most cases, take the place of gum arabic in our extemporaneous emulsions, as it forms a preparation of snowy whiteness.

Castor oil is conveniently taken by suspending it between a little syrup and wine, or an infusion of coffee in a wine glass; and by taking care not to bring the lips together on the glass, no trace of the oil will be left in the mouth.

The concentrated fluid extracts are much superior to the officinal tinctures. I have successfully replaced part of the hydro-alcoholic menstruum in many of the fluid extracts, with sugar; rendering them more agreeable, without detracting from their efficacy as remedial agents. Most of the alcoholic tinctures, might, I think, be successfully treated in the same way.

Strong fruit syrups, prepared by dissolving 24 ounces of refined sugar in a pint of the expressed juice of berries, with gentle heat, make delightful adjuvants to many extemporaneous prescriptions; a small quantity of glycerine should be added to the changeable syrups to prevent them from fomenting. A delightful syrup may be prepared from the essential oil of sweet orange-peel. Muriate of ammonia is best given in syrup of liquorice root; it is preferable to a solution of the black extract—covering the taste equally well, and having the advantage of forming with the salt, a clear mixture. Syrup of wild cherry bark is an excellent adjuvant to the officinal syrup of sarsaparilla, and to cough mixtures. It is a good vehicle for anodynes used in pulmonary complaints. A strong syrup of roasted coffee is an excellent vehicle for administering bitter alkaloids, as quinia, morphia, etc.; they may be conveniently given in powder, by mixing the alkaloid without breaking the crystals, by gently agitating the crystals with a sufficient quantity of impalpable powder of sugar of milk. In this way each crystal is enveloped with the sugar, and when placed upon the tongue and quickly washed down with a draught of water, leaves no taste but the dissolving sugar.

Chloroform nearly covers the taste of bitter alkaloids and many bitter tinctures. It remains to be ascertained whether the properties of these medicines are changed by the presence of chloroform.

Infusion of red rose leaves is a good addition to many extemporaneous mixtures—disguising the taste, and imparting a beautiful color. Chalk mixture should never be prepared with cinnamon water made with oil of cassia—the essential oil of ceylon cinnamon should always be used, as it produces a medicated water of better flavor, and more agreeable odor.

Epsom salts may be made into an agreeable drink with water, sugar, and lemon juice or citric acid. The disagreeable sulphurets are best given in glycerine. Iodine and its salts may be given in glycerine. The solubility of the iodine is facilitated by the addition of a little iodide of potassium. Camphor possesses the property of disguising the penetrating saline taste of iodide of potassium. Escharotics, as iodide and chloride of zinc, nitrate of mercury, etc., in glycerine, are more persistent in their action and easier controlled. Glycerine is a good addition to tannin-containing mixtures; it will dissolve its own weight of tannic acid.

Volatile substances, as ether, chloroform, turpentine, etc., are best given in capsules of gluten; a capsule containing five minims of the medicine, does not weigh over two grains. These capsules are now imported from France, but there is no good reason why they can not be made in our own country.

A solution of cantharadin and pure india-rubber in chloroform, is not attended with the disagreeable contraction of the cantharidal collodion. A little glycerine added to collodion, will keep the gun-cotton flexible when dried on the skin, and prevent it from contracting so strongly as to become cracked.

Cerates and ointments should be of a creamy softness, and when intended to be white, they should be snow-white; to accomplish this, butter of cocoa may be substituted for lard, and glycerine for fats and oil, in most cerates. When practicable, cerates should be delicately perfumed. Sulphur ointment should be colored by the addition of carmine; most persons have a peculiar aversion to its yellow color. A good substitute for sulphur ointment may be made, by boiling together three parts of sulphur, one of caustic soda, and ten parts of water, stirring until the soda and sulphur are united, when the fluid may be decanted and kept from the air; by this process, sulphur is obtained in a fluid form. One application of this mixture, rubbing it into the skin 15 or 20 minutes, has effectually killed the little insects, (*acarus.*) Many powders are best given to children, in a lozenge or *boudon*, delicately flavored and rose-colored. Santonin, as an anthelmintic, should be given this way. Powders may be given enveloped between two thin slices of some fruit jelly—wafer envelopes are less bulky. They may be made by taking a portion of the finest wheaten flour made into a thin paste with water, and baked between two smooth irons pressed firmly together. When removed, it should be cut into a square sheet of convenient size; it is used by dipping into water and dropping the powder into the centre—the edges folded over it, when it may be swallowed like an oyster, without tasting the contents.

Pills should always be coated—it is but the work of a minute, and there is no excuse for obliging our patients to swallow bitter bills. In the last century, pills were frequently coated with gold-leaf; and it is sometimes resorted to at the present day. It is truly a very elegant method, and one that is calculated to please the patient; their solubility and consequent activity I can not answer for; though I conclude, from the extreme thinness of the gold-leaf, that their action is scarcely retarded. Pills are coated with gold-leaf by first moistening them with a little syrup, and introducing them into a hollow sphere along with the requisite quantity of gold-leaf; by rapid motion of the globe for a few seconds the pills are turned out with a gilt coating.

Pills are easily and effectually coated by first moistening with gum paste, and then rolling in finely powdered sugar of milk; the sugar of milk is preferable to common sugar, from being not so easily soluble. The polished coating of the manufacturers can only be obtained by carefully regulated heat and considerable time in the manipulation, and therefore is not practicable for extemporaneous prescriptions. Pills containing salts that are decomposed by exposure to the air, should be coated with an etherial solution of mastic, or just as good, the dried gum of *silphium lancinatum*, the common rosin weed of our prairies. Sulphate of quinia is best made into a mass by adding a sufficient quantity of aromatic sulphuric acid to form a thin paste, and rubbing with a spatula on a tile, until the paste is of proper consistence. No gum or syrup need be added. Pills should be as small as practicable, and consequently the syrup or gum, added to form the mass, should be used as sparingly as possible. Several years ago I prepared granules of a few medicines, to show that allopathic doses can be exhibited in as small form as any one would wish, and still be as effectual as more bulky doses. A case containing five vials of these granules, was no larger than a gentleman's watch, and could be conveniently carried in the vest pocket. The physician often wants medicines in a hurry, and no time can be spent to send to the apothecary. These little cases containing medicines calculated to meet the greatest variety of indications, were used for these special occasions by several of the best physicians of Chicago, and with great satisfaction to their patients. Several instances were related to me, where families of the homœopathic faith, employed the regular practitioner because they heard they gave medicines that were easy to take! As an example of minuteness—the veratrum viride granules contained only the solid matter of one minim of Norwood's tincture, and I think must have been smaller than any pill Hahnemann ever dreamed of. It has been suggested that saturated tinctures of medicinal plants, not easily decomposed by heat, be inspissated upon cane sugar, or sugar of milk, as an eligible form for administration. Homœopathic globules are made of flour and cane sugar; why not make use of the little granules, by medicating them with concentrated alcoholic solutions of the more powerful alkaloids, and while it is the fashion for the doctor to prescribe *something*, whether the patient is sick or not, I apprehend that the *unmedicated* globules would have a happy effect in many cases.

It seems to be desirable to have medicines in as small bulk as possible; for this reason the active principles of plants have gained great favor with the profession. To meet the demand for these favorite medicines, Messrs. Tilden & Co., and Keith & Co., have sent into the market a great variety of so-called *active* principles, which are recognized and prescribed as such, by the best practitioners. These so-called *active* principles, are chiefly dessicated alcoholic extracts, and should not be called by names that can only be properly applied to active principles. This unfortunate ruse will lead to confusion and trouble hereafter, when the *real* active principles are obtained.

---

Valerianate of Ammonia in Neuralgia. — A decided success has been obtained at the Royal Free Hospital, in the treatment of the more severe forms of neuralgia by the valerianate of ammonia, and a number of cases are cited in the *Lancet*, where prompt and permanent relief followed the exhibition of the salt in doses of twenty grains or more. As the crystals are very deliquescent and decompose rapidly, it is recommended that the salt be kept in solution, and given in an infusion of some of the tonic bitters.

The iodide of ammonium is also meeting with much favor in Guy's Hospital, in the treatment of certain glandular swellings,—in bronchocele it is found particularly beneficial, effecting cures after iodine and iodide of potassium had failed. It is given, internally, in five-grain doses, two or three times a day, in some bitter infusion; locally, in the proportions of a drachm to the ounce of glycerine, as a liniment, or of simple cerate, as an ointment.

